# The Bacterial Enzyme Cas13 Interferes with Neurite Outgrowth from Cultured Cortical Neurons

**DOI:** 10.3390/toxins13040262

**Published:** 2021-04-07

**Authors:** Qin-Wei Wu, Josef P. Kapfhammer

**Affiliations:** Department of Biomedicine, Institute of Anatomy, University of Basel, 4056 Basel, Switzerland; qinwei.wu@unibas.ch

**Keywords:** neurotoxic action, Cas13, neuron

## Abstract

The CRISPR-Cas13 system based on a bacterial enzyme has been explored as a powerful new method for RNA manipulation. Due to the high efficiency and specificity of RNA editing/interference achieved by this system, it is currently being developed as a new therapeutic tool for the treatment of neurological and other diseases. However, the safety of this new generation of RNA therapies is still unclear. In this study, we constructed a vector expressing CRISPR-Cas13 under a constitutive neuron-specific promoter. CRISPR-Cas13 from *Leptotrichia wadei* was expressed in primary cultures of mouse cortical neurons. We found that the presence of CRISPR-Cas13 impedes the development of cultured neurons. These results show a neurotoxic action of Cas13 and call for more studies to test for and possibly mitigate the toxic effects of Cas13 enzymes in order to improve CRISPR-Cas13-based tools for RNA targeting.

## 1. Introduction

The CRISPR-associated protein 13 (Cas13) was initially found in bacteria. The Cas13 enzyme contains two higher eukaryote and prokaryote nucleotide-binding domains associated with ribonucleases [1,2,3,4,5]. Since Cas13 was identified, it has exhibited great potential to be utilized as an RNA-guided RNA-targeting CRISPR-Cas effector. The expression of CRISPR-Cas13 together with guide RNA in mammalian and plants cells can achieve RNA editing and knockdown of endogenous transcripts without the production of substantial off-target effects, making the CRISPR-Cas13 platform attractive for therapeutic applications [1,2,3,4]. Within recent years, CRISPR-Cas13-based methods are developing rapidly. More and more studies have explored the use of the CRISPR-Cas13 system in the field of therapeutic agents, including neurological disease [6,7], cancer treatment [8], and antiviral drug development in infectious diseases [9]. If this system was to be used as a tool for RNA manipulation in a therapeutic setting, it is essential that no cellular toxic effects occur. No toxic effects on cellular health by using a Cas13 variant from *Ruminococcus flavefaciens XPD3002* have been found in zebrafish [4]. In contrast, in a recent study, it was reported that the toxicity of this Cas13 protein occurs in fly development [5].

While the CRISPR-Cas13 system certainly is a promising tool for RNA-based therapies in many medical fields, little focus has been put on the safety of this tool. In view of the recent conflicting results on the cellular toxicity of the novel CRISPR-Cas13 protein [4,5], it is of course important to explore the possible dangers of using a Cas13-based platform. In this study, we expressed the bacterial protein CRISPR-Cas13 in primary mouse cortical neurons in culture and checked for possible negative effects of Cas13 expression. Our results demonstrate that the Cas13 protein has a marked neurotoxic effect on the development and differentiation of cultured neurons.

## 2. Results

### 2.1. Neuron-Specific CRISPR-Cas13 Expression in Cortical Neuron Cultures

**Figure 1:**
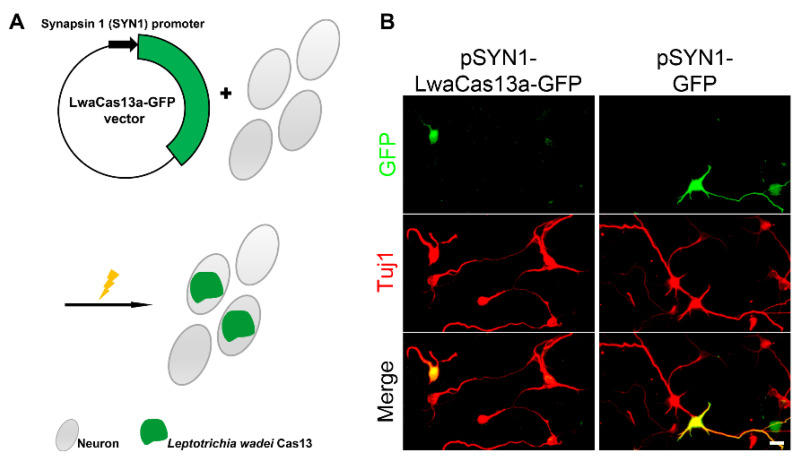
Neuron-specific Cas13 expression. (**A**) A schematic view of the neuron-specific Cas13 expression construct; (**B**) Distribution of LwaCas13a-GFP or GFP expression observed after two days in cultured mouse cortical neurons. LwaCas13a-GFP remains mostly restricted to the cell soma. Scale bar is 20 μm.

In order to achieve neuron-specific expression, we have cloned the engineered GFP fusion LwaCas13a sequence from the Addgene Plasmid #91902 into the pSYN1 vector containing a synapsin 1 (SYN1) neuron specific promoter. Cerebral cortical neurons were maintained in dissociated culture and transfected by electroporation with the pSYN1 vector. With this vector, we achieved neuron-specific expression of the Cas13 protein in neurons by transfection (Figure 1A).

The expression plasmids for LwaCas13a under the control of the SYN1 promoter were transfected after dissociation of the neurons at the day of culture setup. pSYN1-LwaCas13a-GFP was specifically expressed in neurons which were identified by anti-Tuj1 immunostaining as a neuronal marker. Though LwaCas13a was fused to GFP, we found that the expression of LwaCas13a-GFP was restricted to the cell soma and only few processes were seen. This was in contrast to GFP-expression alone from the same pSYN1 vector, which was strongly expressed in the cell body and the neurites of the neurons (Figure 1B).

### 2.2. CRISPR-Cas13 Impedes Development of Cultured Neurons

**Figure 2:**
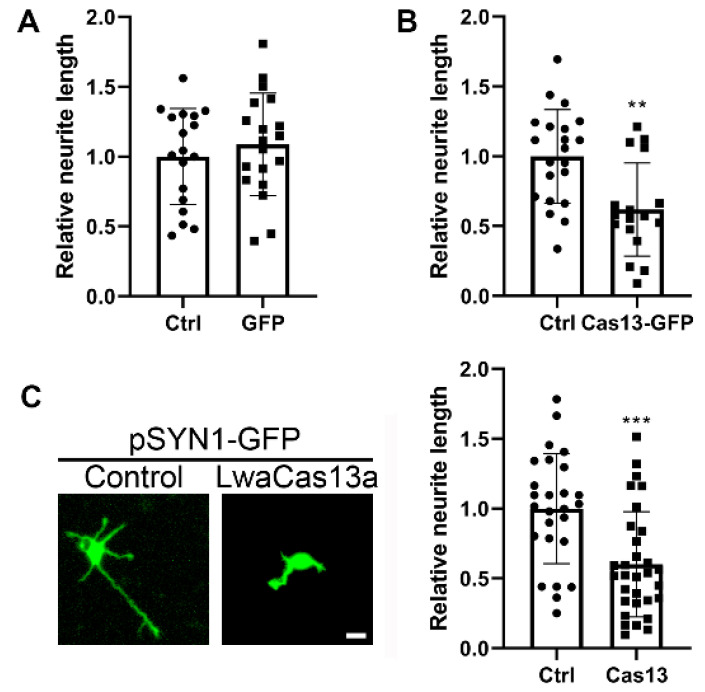
Neuron-specific Cas13 expression impedes neuronal development. (**A**) Neurite length of neurons transfected with pSYN1-GFP was normal compared to non-transfected cells in the same culture well. The *n* of non-transfected cells vs. GFP was 18 vs. 19, the mean was 1.00 ± 0.344 vs. 1.09 ± 0.366. The absolute mean value of neurite length in non-transfected cells (Ctr) was 214.49 ± 73.732 μm. (**B**) Neurite length of neurons transfected with pSYN1-LwaCas13a-GFP was strongly reduced. The *n* of non-transfected cells vs. LwaCas13a-GFP was 21 vs. 17, the means were 1.00 ± 0.334 vs. 0.62 ± 0.333, the absolute mean value of neurite length in non-transfected cells (Ctr) was 205.68 ± 68.687 μm. The result was significant with ** *p* = 0.0012 in the two-tailed Mann–Whitney test. (**C**) A significant difference in the neurite length of neurons was also observed for the transfection of pSYN1-LwaCas13a-GFP together with pSYN1-GFP vs. the transfection of pSYN1-GFP alone. *n* of control = 26 and *n* of LwaCas13a = 31 from four independent culture wells. The mean of the control was 1.00 ± 0.394, the mean of LwaCas13a was 0.60 ± 0.376. The result was significant with *** *p* = 0.0005 in the two-tailed Mann–Whitney test. The values shown are the mean ± SD. Scale bar is 20 μm.

On inspection of the cultures, it was evident that GFP expression alone did not affect the process outgrowth of transfected neurons (Figure 1B, right side), but the expression of LwaCas13a-GFP clearly reduced the length of the neuronal processes (Figure 1B, left side). In a second step, the total length of the neurites of neurons was measured, and it was confirmed that GFP expression by itself did not significantly affect neurite length compared to control non-transfected GFP-negative neurons in the same culture well (Figure 2A). However, we found that the length of the neurites of LwaCas13a-GFP transfected neurons was reduced to 62% (*p* = 0.0012) of control neurons (Figure 2B). We also co-transfected pSYN1-GFP and pSYN1-LwaCas13a-GFP with a mass ratio of 0.2–0.5 and compared the morphology to the control group transfected with pSYN1-GFP alone in order to better see the morphology of single neurons. After transfection, the length of the neurites of pSYN1-LwaCas13a-GFP transfected neurons was again reduced to 60% (*p* = 0.0005) of pSYN1-GFP transfected control neurons (Figure 2C). This experiment confirmed that LwaCas13a has a negative impact on neuronal development with a marked reduction in neurite length.

## 3. Discussion

In this study, we demonstrate that expressing the CRISPR-Cas13 protein in mouse cerebral cortical neurons results in reduced neurite growth and development, suggesting the presence of a neurotoxic action from the CRISPR-Cas13 protein. In recent studies, potential toxic effects of the Cas13 variant from *Leptotrichia wadei* (LwaCas13a) have been investigated during zebrafish embryonic development. However, LwaCas13a-GFP showed poor expression in zebrafish embryos and possible toxic effects could not be tested [4]. The Cas13 protein from *Ruminococcus flavefaciens XPD3002* (RfxCas13d) was expressed and no evidence for embryonic toxicity was found [4]. In another recent study, however, it was found to have a toxic effect in embryonic flies when expressed under a tissue specific promoter [5]. These conflicting results could be explained if the toxic effects of Cas13d were specific to *Drosophila*, or that a sustained RfxCas13d expression under a strong or constitutive promoter may be required for causing toxicity [4]. We have found earlier that LwaCas13a expression has a negative impact on Purkinje cell development [3,10,11], and *Porphyromonas gulae* (PguCas13b) and *Prevotella sp. P5-125* (PspCas13b) have been reported to have a negative impact on zebrafish embryonic development [2,4]. We now show that a sustained Cas13 expression by a constitutive neuron-specific promoter SYN1 produced a neurotoxic action in cortical neurons. Evidence from this study and recent studies shows that CRISPR-Cas13 variants have cellular toxic effects in zebrafish, fly or mouse models. Our findings also corroborate recent studies suggesting that potential cellular toxic effects of Cas13 may occur in particular during developmental stages.

The approval by the US Food and Drug Administration (FDA) of the first RNA interference therapy opens a path for future RNA treatments in human patients, and some companies might seek for approval of Cas13-based RNA interference technologies for clinical treatments of genetic diseases soon [12]. RfxCas13d has also recently been studied in vivo for future therapeutic use in neurological diseases [6,7]. We should be aware that negative information about new Cas13-based RNA technology may be easily overlooked in the case of exciting positive results. Therefore, our findings are a reminder to pay more attention to potential negative side effects of Cas13 and to investigate the mechanisms of these toxic properties in order to make future clinical use of this technology possible without any danger of potential risks.

## 4. Materials and Methods

### 4.1. Mice

All experiments were carried out in accordance with the EU Directive 2010/63/EU for the care and use of laboratory animals, were permitted by Swiss authorities and approved by the veterinary office of the canton of Basel-Stadt (ethical approval code: 1708; ethical approval date: 3 December 2019, valid through 31 December 2022). FVB mice were used for primary mouse cerebral cortical neuron cultures.

### 4.2. Immunocytochemistry

Primary mouse cerebral cortical neurons were fixed in 4% paraformaldehyde for 30 min at room temperature. All reagents were diluted in 100 mM phosphate buffer (PB), pH 7.3. Fixed cells were incubated with primary antibody diluted in blocking solution (PB + 3% non-immune goat serum + 0.5% Triton X-100) for 1 h at room temperature. After washing with PB, the corresponding fluorescence-conjugated secondary antibodies were added to the cells in PB containing 0.1% Triton X-100 for 2 h at room temperature. The following primary antibodies were used: mouse monoclonal anti-β-Tubulin III (Tuj1) antibody (1:1000, Sigma-Aldrich, Buchs, Switzerland, category number T8660); rabbit anti-GFP (1:2000, Novus, Zug, Switzerland); the staining was visualized with anti-mouse Alexa 568 and anti-rabbit Alexa 488 secondary antibodies (1:2000, Molecular Probes, Eugene, OR, USA); the stained cells were mounted with Mowiol (Sigma-Aldrich, Buchs, Switzerland). The images were captured on an Olympus AX-70 fluorescence microscope equipped with a Spot Insight digital camera.

### 4.3. Plasmid Construction

Engineered Cas13 coding sequence was cloned into the expression plasmid pSYN1 vector for cell-specific expression in neurons and prepared using the EndoFree Plasmid Maxi Kit (QIAGEN, Hilden, Germany) according to the manufacturer’s protocol. Engineered Cas13 sequence was PCR amplified from pC014-LwCas13a-msfGFP, Addgene #91902, a gift from Feng Zhang.

### 4.4. Cultures of Primary Cortical Neurons and Transfection Procedure

Primary mouse cortical neuron cultures were prepared from neonatal mice according to the optimized protocol for primary mouse hippocampal and cortical neurons from Lonza. Briefly, brains from postnatal day 0 mice were dissected and plated on glass chambers coated with Poly-d-lysine. pSYN1-LwaCas13a-GFP vectors were introduced into neurons by transfection with a Nucleofector 2b Device using the program O-005. Cells were incubated in an appropriate volume of culture medium I (Dulbecco’s Modified Eagle Medium, DMEM supplemented with 10% Fetal Bovine Serum, FBS). At 2–4 h after transfection, an equal volume of culture medium II (Neurobasal supplemented with 2% B27 supplement and 2 mM GlutaMAX) was added to each well. After 24 h the medium was replaced with fresh culture medium II. The media and supplements were from Life Technologies, Zug, Switzerland. Cells were kept in culture for two days before fixation.

### 4.5. Quantitative Analysis

The quantification of neurite length was performed by an image analysis program (ImageJ). The average value of control neurons was set as 1. In order to ensure a comparable growth environment, GFP negative neurons close to GFP positive neurons from the same well were taken as control in this study. The shown images were linearly adjusted in brightness and contrast. The data were analyzed using GraphPad Prism software (San Diego, CA, USA). The statistical significance of differences in parameters was assessed by nonparametric Mann–Whitney’s test. Confidence intervals were 95%, statistical significance was assumed with *p* < 0.05.

## Data Availability

The original data are available upon request from the authors.
